# pH-activated antibiofilm strategies for controlling dental caries

**DOI:** 10.3389/fcimb.2023.1130506

**Published:** 2023-03-06

**Authors:** Xiuqing Wang, Jingling Li, Shujun Zhang, Wen Zhou, Linglin Zhang, Xiaojing Huang

**Affiliations:** ^1^ Fujian Key Laboratory of Oral Diseases & Fujian Provincial Engineering Research Center of Oral Biomaterial & Stomatological Key Lab of Fujian College and University, School and Hospital of Stomatology, Fujian Medical University, Fuzhou, China; ^2^ State Key Laboratory of Oral Diseases, Department of Cariology and Endodontics, National Clinical Research Center for Oral Diseases, West China Hospital of Stomatology, Sichuan University, Chengdu, China

**Keywords:** biofilm, pH-responsive, antibiofilm agents, drug delivery systems, dental caries, *Streptococcus mutans*

## Abstract

Dental biofilms are highly assembled microbial communities surrounded by an extracellular matrix, which protects the resident microbes. The microbes, including commensal bacteria and opportunistic pathogens, coexist with each other to maintain relative balance under healthy conditions. However, under hostile conditions such as sugar intake and poor oral care, biofilms can generate excessive acids. Prolonged low pH in biofilm increases proportions of acidogenic and aciduric microbes, which breaks the ecological equilibrium and finally causes dental caries. Given the complexity of oral microenvironment, controlling the acidic biofilms using antimicrobials that are activated at low pH could be a desirable approach to control dental caries. Therefore, recent researches have focused on designing novel kinds of pH-activated strategies, including pH-responsive antimicrobial agents and pH-sensitive drug delivery systems. These agents exert antibacterial properties only under low pH conditions, so they are able to disrupt acidic biofilms without breaking the neutral microenvironment and biodiversity in the mouth. The mechanisms of low pH activation are mainly based on protonation and deprotonation reactions, acids labile linkages, and H^+^-triggered reactive oxygen species production. This review summarized pH-activated antibiofilm strategies to control dental caries, concentrating on their effect, mechanisms of action, and biocompatibility, as well as the limitation of current research and the prospects for future study.

## 1 Introduction

Oral biofilms are highly assembled microbial communities surrounded by an extracellular matrix (Bowen et al., 2018). After saliva glycoproteins cling to the tooth surface, oral microorganisms begin to gather and adhere, and form an orderly structured community wrapped in the extracellular matrix (Marsh et al., 2016). Under normal conditions, the resident microbes, including commensal bacteria and opportunistic pathogens, coexist with each other to maintain relative dynamic balance, which plays an essential role in oral and whole-body health (Gao et al., 2018; Rosier et al., 2018). However, under hostile conditions such as high-carbonhydrate diet and poor oral hygiene, glycometabolic microorganisms in the biofilms can generate excessive organic acids from diet through a fermentation process (Simón-Soro and Mira, 2015; Hajishengallis et al., 2017). The organic acids are confined in the local biofilm by extracellular matrix, creating acidic niches (Benoit et al., 2019). The acidic environment affects the metabolic activity of oral microorganisms and exhibits an acid-induced selection, increasing the proportions of acidogenic and aciduric bacteria, such as *Streptococcus mutans* (*S. mutans*), the major cariogenic bacteria (Takahashi and Nyvad, 2011). Dental caries start with the break of the oral eubiosis, shifting from commensal biofilm to cariogenic biofilm with abundant acidogenic and aciduric microbes (Selwitz et al., 2007; Pitts et al., 2017; Tanner et al., 2018). There is a prolonged drop in pH value of cariogenic biofilm, leading to demineralization of dental hard tissue and development of dental caries when the value falls below 5.5 and lower (Margolis et al., 1999; Selwitz et al., 2007; Pitts et al., 2017; Xu et al., 2022b). Study confirmed that the pH at active caries niches was reduced to about 4.5 - 5.5 (Bowen, 2013). Therefore, it is tremendous significance to control acidic biofilm to prevent and treat dental caries.

Despite remarkable progress in the prevention of dental caries, especially with the application of fluoride, controlling acidic dental biofilms is still associated with serious challenges (Liu et al., 2018). Compared with planktonic bacteria, pathogens in the established biofilm are protected by extracellular matrix barriers (Kuang et al., 2018). Conventional drugs are incapable of degrading the matrix, resulting in far less effective against preformed acidic biofilm. Moreover, conventional broad-spectrum antimicrobials, such as chlorhexidine (CHX), kill oral microorganism effectively without selectivity, bringing challenges to long-term therapeutic use (Zhang et al., 2022a). Frequent use of antibacterial strategies without selectivity would potentially damage the ecological balance, which increases possibility of reinfection by opportunistic pathogens (Guo et al., 2015).

Efforts have been made to develop novel strategies to deal with these problems, including the following: 1) killing cariogenic pathogens specifically by *S. mutans*-specific targeting antimicrobial agents (Guo et al., 2015; Huo et al., 2018; Xiang et al., 2019); 2) modulating the biofilm pH *via* alkali production by alkali-producing bacteria to protect against plaque acidification and further dominance of cariogenic bacteria that thrive in acidic conditions (Liu et al., 2012); 3) digesting the protective extracellular matrix *via* enzymes to facilitate penetrability of antibacterial agent into mature biofilms (Gao et al., 2016; Liu et al., 2016); and 4) activating antimicrobial capacity smartly by components that can be activated by ambient stimuli (Gupta et al., 2002; Kost and Langer, 2012). Various stimuli are applied in stimuli-triggered antimicrobial strategies, such as thermal (Gopal et al., 2016; Imai et al., 2018), pressure (Montoya et al., 2021) and pH (Chen et al., 2022). pH is closely related to caries development, that is, dental hard tissues demineralize once the ambient pH continuously drops below 5.5. As mentioned above, the pH of local niches of caries is about 4.5 - 5.5, while in the physiological conditions, salivary pH range 6.2 - 7.6 (Baliga et al., 2013). Therefore, acidic pH is the most promising stimuli that can be used to combat cariogenic biofilm smartly. Acidic-triggered strategies show antibacterial effect only under acidic conditions and do not function under neutral physiological conditions, exhibiting ability to maintain oral microecological homeostasis (Liu et al., 2018; Liang et al., 2020). In recent years, acidic-triggered strategies have attracted more and more attention in the prevention and treatment of caries, based on keeping balance and biodiversity of oral microecology (Liang et al., 2020; Naksagoon et al., 2021). This review summarizes the research progress of pH-responsive antibiofilm strategies to control dental caires in recent years, mainly focusing on antibiofilm effect, antimicrobial mechanism and biocompatibility, as well as the limitation of current research and the prospects for future research, in an attempt to provide reference for subsequent study.

## 2 pH-responsive antibiofilm strategies

The pH-activated antibiofilm strategy enhances the selectivity and efficacy of antimicrobial agents. Recent researches have focused on designing novel pH-activated strategies to control dental caries, including pH-responsive antimicrobial agents and pH-sensitive drug delivery systems.

### 2.1 pH-responsive antibiofilm agents

Several pH-responsive antibiofilm agents have been generated to inhibit the formation and development of cariogenic biofilm, and disrupt the unbalanced microbial composition. The agents include: 1) pH-responsive antimicrobial peptides; 2) organic compounds with amine groups; 3) iron oxide nanoparticles with peroxidase-like activity (**Table 1**).

**Table 1:** pH-responsive antibiofilm agents to control acidic biofilm.

Class	pH responsive antibiofilm agents	Antibiofilm activity	Mechanisms of action	Toxicity	Assay	Author, year
**Antimicrobial peptide**	Histatins-5	Inhibit the formation of *S. mutans* biofilm	Protonation of histidine residues under acid environment	–	*In vitro*	(Mochon and Liu, 2008; Krzyściak et al., 2015)
**Antimicrobial peptide**	Kappacin	Inhibit the growth of *S. mutans*, *P. gingivalis* and *A. naeslundii*	Membranolytic action at acidic pH	–	*In vitro*	(Malkoski et al., 2001; Dashper et al., 2007)
**Antibacterial peptide nanoparticles**	pHly-1 NPs	Inhibit formation of EPS and *S. mutans* biofilm and development of *S. mutans* biofilm	*Via* protonation of the histidine at low pH, peptide pHly-1 adopts random coil-helix conformation at low pH and forms nanoparticles	Low toxicity on the normal oral and gastric tissues	*In vitro* & *in vivo*	(Zhang et al., 2022a)
**Antimicrobial peptide**	AAPs	Inhibit the growth of *S. mutans* within the biofilm community	*Via* protonation of histidine residues at low pH, AAPs undergo a transition from a helical conformation to a random coil	–	*In vitro*	(Clark et al., 2021)
**Antimicrobial peptide**	GH12	Inhibit formation of EPS, water-insoluble glucan, and lactic acid in *S. mutans* biofilm as well as killing *S. mutans* within the multispecies biofilm	Due to protonation of histidine residues under acid environment, GH12 forms an amphipathic α-helix	Negligible cytotoxicity to human gingival fibroblast cells	*In vitro*	(Tu et al., 2016; Wang et al., 2017; Jiang et al., 2018; Jiang et al., 2021)
**Antimicrobial peptide**	LH12	Inhibit virulence and growth of cariogenic pathogens, as well as enhancing the competitiveness of commercial bacteria in the mixed-species microbiota	Via protonation of histidine residues under acid environment, LH12 forms an amphipathic α-helix	Show slight cytotoxicity to human gingival epithelial cells at a high concentration of 128 μg/mL	*In vitro*	(Jiang et al., 2022)
**Quaternary pyridinium salt**	Azo-QPS-C16	Inhibit acid-producing bacteria in multispecies biofilm	Weakly acid Azo-QPS-C16 and bases can assemble into inactive agglomerate at neutral pH, but the agglomerate will collapse at low pH	–	*In vitro*	(Yang et al., 2018)
**Tertiary amine**	DMAEM HMAEM	Inhibit formation of EPS and *S. mutans* biofilm and regulate oral microecological balance	Protonation of amine groups in tertiary amine under acid environment	Low cytotoxicity to human oral keratinocyte cells	*In vitro* & *in vivo*	(Liang et al., 2020; Shi et al., 2022)
**Nanoparticles**	Iron oxide nanoparticles	Kill bacteria in biofilm and break down EPS	The peroxidase-like activity of iron oxide nanoparticles	High biocompatibility	*In vitro* & *in vivo*	(Gao et al., 2016)

#### 2.1.1 pH-responsive antimicrobial peptides

Antimicrobial peptides (AMPs), a kind of pervasive natural peptides, exert the ability of antibiosis and antivirus and are present in both plants and animals as potent antibiotics for the inherent immune system (Zasloff, 2002; Reddy et al., 2004; Harris et al., 2009). These peptides have been reported to show the ability to inhibit the formation and development of pathogenic biofilm (Luo and Song, 2021), indicating that AMPs may be a promising antibiofilm strategy for dental caries control. Besides, AMPs are less likely to induce drug resistance since they target almost non-specific modes at multiple sites on microbial membranes (Malik et al., 2016; Steinbuch and Fridman, 2016). However, non-specific targeting to oral microorganism would potentially lead to ecological dysbiosis, which increases possibility of opportunistic infections (Eckert et al., 2006). In order to increase selectivity of AMPs, researchers developed novel kinds of AMPs with a targeting domain and an antimicrobial domain, such as C16G2 and C10-KKWW, which can selectively target *S. mutans* and kill them effectively (Guo et al., 2015; Xiang et al., 2019). These peptides exert efficacy in not only cariogenic but also healthy niches. Another strategy is pH-activated AMPs, which exert potent antimicrobial efficiency only in acidic environment and do not function under neutral physiological conditions, exhibiting ability to maintain oral microecological homeostasis (Liu et al., 2018; Liang et al., 2020). Some natural peptides have pH-responsive properties, and a number of researchers focus on designing novel pH-activated antimicrobial peptides.

Histatins, isolated from the human parotid salivary gland in 1988, is a group of pH-activated peptides rich in histidine residues (Fabian et al., 2012; Krzyściak et al., 2015; Khurshid et al., 2017). The antifungal activity of histatin-3 and histatin-5 have been proven to be enhanced by acidic pH (Mochon and Liu, 2008), and histatin-5 exhibits the ability to inhibit the formation of *S. mutans* biofilm (Krzyściak et al., 2015). Kappacin, another natural antibacterial peptide, is the active form of Caseinomacropeptide, a heterogeneous C-terminal fragment from bovine milk (Malkoski et al., 2001). Kappacin has been proven to exhibit the antibacterial activity of inhibiting the growth of Gram-negative and Gram-positive bacteria in oral cavity, including *S. mutans*, *Porphyromonas gingivalis* (*P. gingivalis*) and *Actinomyces naeslundii* (*A. naeslundii*),which are components of supra gingival dental plaque (Malkoski et al., 2001; Dashper et al., 2007). An increase in antimicrobial activity against *S. mutans* and *A. naeslundii* has discovered under mildly acidic conditions.

Peptide pHly-1 forms into nanofibers at physiological pH, but can generate coil-helix conformation and turn into nanoparticles under an acid environment (Zhang et al., 2022a). It has been confirmed that pHly-1 nanoparticles were capable of suppressing both formation and development of *S. mutans* biofilm at acidic pH, while negligible antibiofilm activity was found at neutral pH. The minimum inhibition concentration (MIC) and minimum bactericidal concentration (MBC) of pHly-1 nanoparticles against *S. mutans* were estimated to be 5.5 μM and 6.7 μM at pH 5.5, but > 44 μM and > 22 μM at pH 7.0, respectively. Besides, the agent exerted potent effects in inhibiting bacterial clusters and the formation of extracellular polymeric substances (EPS) in the preformed biofilms at pH 4.5 while negligible effects were observed at pH 7. Furthermore, initial, moderate, and severe dental caries lesions were significantly decreased by the use of pHly-1 NPs in the animal study with a rat carious model. It is worth mentioning that pHly-1 NPs exhibited a better anticaries effect than CHX *in vivo*.

Clavanins are a kind of a-helical amphipathic antimicrobial peptide with 23 amino acid residues, which were firstly purified from hemocytes of the invertebrate styela clava. It was found that Clavanin A was rich in both histidine and phenylalanine residues and had broad-spectrum antibacterial properties (Lee et al., 1997b). When the pH drops, the protonation of histidine residues enhances the ability to target bacterial membranes. At the same time, phenylalanine residues enable the AMP to form a flexible and hydrophobic structure to facilitate the interaction with membrane lipids (Lee et al., 1997a; van Kan et al., 2002; van Kan et al., 2003a; van Kan et al., 2003b). According to the structure of Clavanin A, two acid-activated peptides (AAPs), named AAP1 and AAP2, were designed to combat dental caries (Clark et al., 2021). It appeared that AAPs performed a more potent antibacterial ability than Clavanin A under a low pH value while overcoming Clavanin A’s shortcoming of exhibiting antimicrobial efficacy at neutral pH. APP2 exerted more potent antibacterial activity at pH 5 than AAP1. In the test against microbes in *S. mutans* biofilms, AAP2 showed the potential to reduce the acid-producing flora within the biofilm community.

Inspired by the template (XXYY)n (X refers to a hydrophobic residue, Y refers to a hydrophilic residue, and n refers to the number of repeats) proposed by Wiradharma et al. as an appropriate structure of amphipathic, cationic α-helical AMPs, Wang et al. designed a peptide with 12 amino acid residues called GH12 against cariogenic bacteria (Wiradharma et al., 2011; Wang et al., 2017). Study showed that GH12 had much lower MIC against dental caries-associated bacteria (*S. mutans*, *Streptococcus sanguinis*, *Streptococcus gordonii* (*S. gordonii*), *Streptococcus mitis*, *Streptococcus salivarius*, *Streptococcus sobrinus*, *Actinomyces viscosus*, *Actinomyces naeslundii*, *Lactobacillus acidophilus*, *Lactobacillus casei*, and *Lactobacillus fermentum*) at pH 5.5 than at pH 7.2. The production of EPS component, water-insoluble glucan synthesis, and lactic acid in preformed *S. mutans* biofilm was also inhibited by GH12, and the inhibitory effects increased when pH dropped from 7.2 to 5.5 (Jiang et al., 2021). In addition to suppressing the formation and viability of *S. mutans* biofilm at pH 5.5, GH12 exhibited different antibacterial effect in killing different bacterial species in multispecies biofilm, which indicates that GH12 have the ability to change the microbiota composition of cariogenic biofilm (Jiang et al., 2018).

Another cationic amphiphilic α-helical AMP, named LH12, was also designed based on the (XXYY)n formula (Jiang et al., 2022). LH12 exhibited stronger antimicrobial activity against *S. mutans* in response to acidic environment. MIC and MBC of LH12 against *S. mutans* were about 12μg/ml and 16μg/ml at pH 5.5, but 21μg/ml and 32μg/ml at pH 7.2. In addition to kill *S. mutans*, 16 μg/mL and 32 μg/mL LH12 could reduce the production of lactic acid and EPS, as well as completely suppressing the biofilm formation. Furthermore, in the dual-species biofilm model, the proportion of *S. gordonii* was increased while the proportion of *S. mutans* was decreased, which indicated that LH12 could regulate the microbial composition.

#### 2.1.2 Organic compounds with amine groups

A novel quaternary pyridinium salt with pH-controlled activity, (E)-1-hexadecyl-4-((4-(methacryloyloxy)phenyl)diazenyl)-pyridinium bromide (Azo-QPS-C16), was designed to curb the growth of acid-producing microbes (Yang et al., 2018). An 8-fold difference in efficacy against *S.mutans* was observed in acidic solutions than in neutral solutions. *Via* the saliva-derived multispecies biofilm model containing *Enterobacter* spp., *Klebsiella* spp. and *Streptococcus* spp., the ability of Azo-QPS-C16 to kill or inhibit acid-producing bacteria was monitored. The result showed that Azo-QPS-C16 could selectively eliminated sucrose-fermenting, acidogenic bacteria in biofilm while increasing the biomass of commensals. It is worth noting that the application of Azo-QPS-C16 was able to maintain the pH of culturing solutions above 5.5, below which demineralization of dental enamel happens (Kleinberg, 2002).

Liang et al. designed and synthesized two kinds of tertiary amine (TA) monomers: DMAEM (dodecylmethylaminoethyl methacrylate) and HMAEM (hexadecylmethylaminoethyl methacrylate). The MICs of DMAEM and HMAEM against the *Streptococci* species ranged from 0.18 to 5.95 μg/mL and 0.2 to 0.8 μg/mL, respectively at pH 5.0, while no antibacterial effect was detected even at the concentration up to 13.5 mg/ml at pH 7.0. Aimed at inhibiting secondary caries, the TAs with pH responsiveness were incorporated into adhesive resins, getting the TA-modified resin adhesives (TA@RAs) (Liang et al., 2020). There was no significant difference in antibiofilm activity between DMAEM@RAs and HMAEM@RAs. However, the pH of DMAEM@RAs and HMAEM@RAs when they started to exert antibacterial efficacy was 5.3 ± 0.03 and 4.1 ± 0.01, respectively, indicating that DMAEM-modified resin adhesives are more sensitive to pH than resin adhesives modified by HMAEM. Researchers in the same group further explored the antimicrobial effect of DMAEA@RA on dual-species biofilms of *S. mutans* and *Candida albicans* to prevent secondary caries (Shi et al., 2022). Results showed that DMAEM@RA were capable of inhibiting the development of dual-species biofilms as well as suppressing the production of EPS and acid when pH was below 5.5, while those activities at pH 6.0 were similar to negative control groups. *Via* down-regulating the expression of pH-regulated genes, virulence-associated, and cariogenic genes, DMAEA@RAs could reduce the mineral loss of teeth both *in vitro* and *in vivo* in a pH-dependent manner.

#### 2.1.3 Iron oxide nanoparticles with peroxidase-like activity

Iron oxide nanoparticles, having been regarded as nanozymes, exert intrinsic enzyme mimetic efficiency to activate H_2_O_2_ which is similar to peroxidases (Gao et al., 2007). The nanoparticles have attracted great attention due to their antibacterial, antifungal, and anticancer abilities and low toxicity to human body (Dadfar et al., 2019). Gao et al. synthesized catalytic iron oxide nanoparticles (CAT-NP) containing Fe_3_O_4_ and found that there was an increase in catalytic efficiency of CAT-NP when pH dropped from 6.5 to 4.5 (Gao et al., 2016). CAT-NP exhibited potent efficacy to induce extracellular matrix degradation and kill bacteria within the acidic microenvironment of cariogenic biofilm. Moreover, it possessed an additional pH-dependent mechanism to control dental caries by directly decreasing the demineralization of enamel in acidic environment. A kind of dextran-coated iron oxide nanoparticles termed nanozymes (Dex-NZM) was designed to specifically target biofilms (Naha et al., 2019). Dextran, a polysaccharide with low toxicity, can be embedded into the matrix of growing biofilms by bacterial exoenzymes, resulting in high selectivity toward biofilms (Gibbons and Banghart, 1967; Xiao et al., 2012). Compared with uncoated NZM, Dex-NZM displayed a better role in controlling dental biofilms at pH 4.5. To go a step further, Huang et al. combined glucose oxidase (GOx) with dextran-coated iron oxide nanoparticles (Dex-IONP) (Huang et al., 2021). GOx can catalyze glucose in cariogenic biofilms to generate H_2_O_2_, which facilitates the pH-dependent production of reactive oxygen species (ROS) by Dex-IONP. Dex-IONP-GOx displayed greater catalytic activity at pH 4.5 and 5.5 than at pH 6.5. In the *in vitro* test with a mixed-species biofilm model, Dex-IONP-GOx was confirmed to inhibit the cariogenic *S. mutans* potently, but with negligible effects against the commensal *Streptococcus oralis*.

### 2.2 pH-responsive drug delivery systems

**Figure 1:**
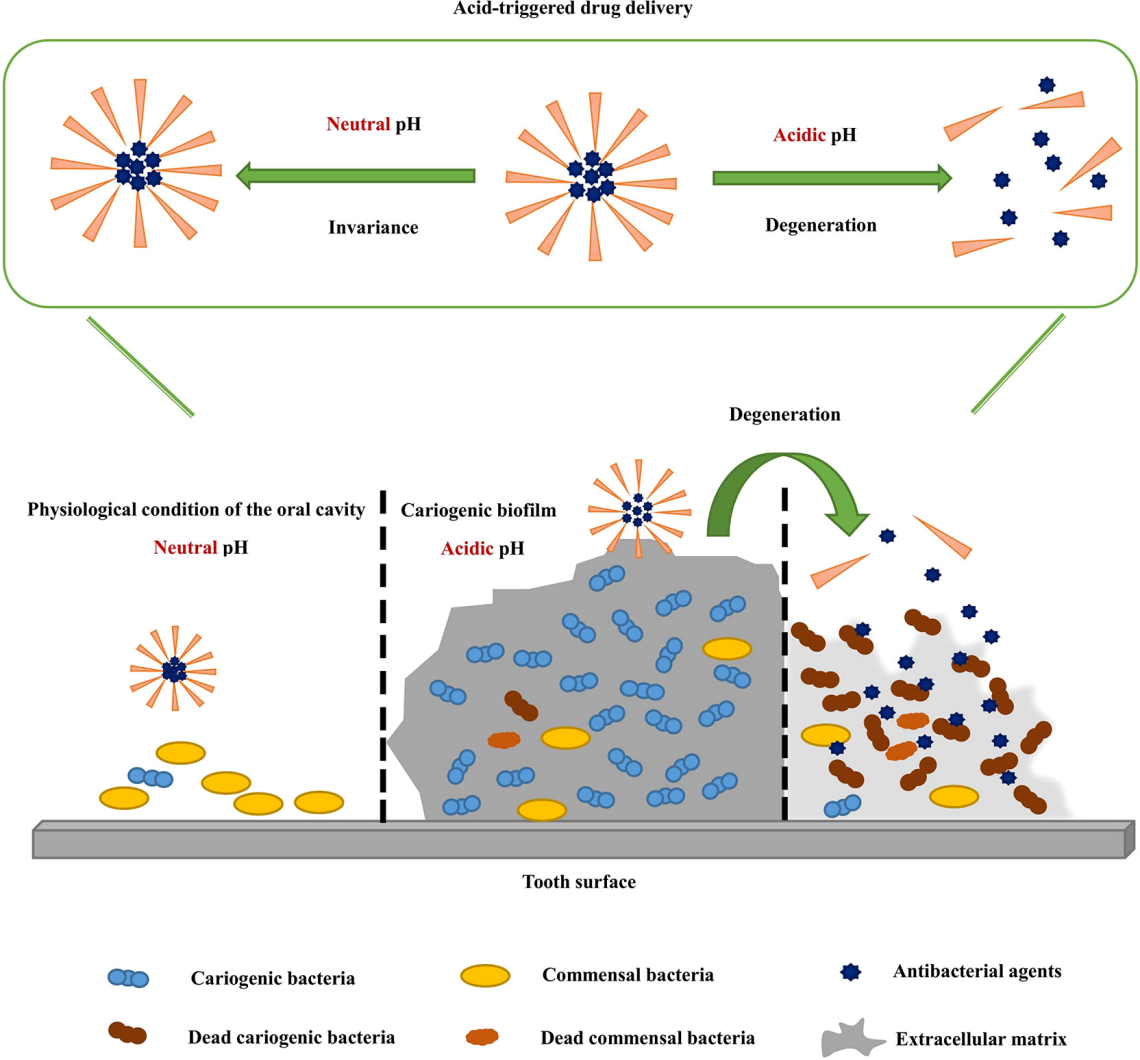
Acid-triggered drug delivery systems deliver the drug to acidogenic biofilms effectively.

The downside of traditional antimicrobial drugs, such as fanasol and CHX, is the toxicity or side effects caused by low selectivity. Killing microorganisms without selectivity reduces the diversity of microbial communities, thus destroying the ecological balance of microbial communities and bringing great challenges for clinical treatment. Therefore, great importance has been attached to strategies by which drugs are delivered without disrupting the internal oral environment. Acid-triggered drug delivery systems are able to deliver the drug to acidogenic biofilms effectively without disrupting the commensal biofilms. The carriers used to fabricate pH-activated drug release systems often contain a specific functional group, which can respond to changes in the pH of the ambient environment. The mechanisms by which carriers respond to pH mainly includes the charge shifting of pH-responsive residues and the degradation of acid-degradable residues (Deirram et al., 2019) (**Figure 1**; **Table 2**).

**Table 2:** pH-responsive drug delivery systems to control acidic biofilm.

Nanocarriers	Drug	Antibiofilm activity	Mechanisms of action	Toxicity	Assay	Author, year
**p(DMAEMA)-b-p(DMAEMA-co-BMA-co-PAA)**	Farnesol	Nanoparticles generate high binding affinities to pellicle and EPS surfaces and enhance antibiofilm activity of farnesol	Protonation of tertiary amines in DMAEMA and carboxyl groups in PAA at acidic pH	–	*In vitro* & *in vivo*	(Horev et al., 2015; Zhou et al., 2016; Sims et al., 2018)
**TMC-Lip nanoparticles**	Doxycycline	Nanoparticles disrupt the biofilm architecture and reduce the number of bacteria significantly	Protonation of amino groups	Good cytocompatibility on human periodontal ligament fibroblasts	*In vitro* & *in vivo*	(Zhou et al., 2018; Hu et al., 2019)
**FePAgPG**	Ag	Nanoparticles inhibit the formation of *S. mutans* biofilm	Protonation of amino groups in glycol chitosan	Good biocompatibility on human oral keratinocytes cells	*In vitro*	(Xu et al., 2022a)
**p(CBMA-co-DMAEMA)**	pH-switchable antibacterial octapeptides	The positively charged surface is able to capture cariogenic bacteria under acid environment and when pH decrease, the number of dead bacteria on hydrogel layers increase	Protonation of carboxyl groups in CMBA and tertiary amines in DMAEMA at low pH	Good biocompatibility on human oral keratinocyte cells	*In vitro*	(Sun et al., 2022)
**poly(DMAEMA-co-HEMA)**	Chlorhexidine	The whole system exhibits the same antimicrobial activities as free chlorhexidine on cariogenic biofilm	Protonation of tertiary amines in DMAEMA at low pH	Lower the cytotoxicity of chlorhexidine against human oral keratinocytes	*In vitro*	(Peng et al., 2022)
**mPEG-b-PDPA**	Bedaquiline	Inhibit *S. mutans* biofilm formation	Protonation of amine groups in PDPA segments under acid environment	No significant cytotoxicity	*In vitro*	(Zhang et al., 2021)
**PEG-b-PAECOEMA/CA**	Chlorhexidine	Respond to acid microenvironments of cariogenic biofilms and rapidly release chlorhexidine for efficient bacteria killing.	Degradation of citraconic amide groups at low pH	The carrier reduces the toxic of chlorhexidine	*In vitro*	(Zhao et al., 2019)
**MAL-PEG-b-PLL/PBA**	Tannic acid & sodium fluoride	PMs@NaF-SAP can suppress the growth of *S. mutans* biofilm and inhibit demineralization and facilitates remineralization in enamel slices	Cleavage of boronate ester bonds	The system exhibits much lower cytotoxicity than chlorhexidine	*In vitro* & *in vivo*	(Xu et al., 2022b)
**PPi-Far-PMs**	Farnesal	PPi-Far-PMs can inhibit the growth of *S. mutans* both *in vivo* and *in vitro*	Cleavage of hydrazone bonds	–	*In vitro* & *in vivo*	(Yi et al., 2020)
**MSNs**	Ag ions & chlorhexidine	Inhibit growth of *S. mutans* and *S. mutans* biofilm formation	Cleavage of disulfide bonds	Significantly reduce the toxicity of chlorhexidine	*In vitro* & *in vivo*	(Yue et al., 2018)
**ZnO_2_-Cu@RB NPs**	Rose Bengal	Kill *S. mutans* in cariogenic biofilm and suppress the formation of EPS	Fenton reaction	–	*In vitro*	(Zhang et al., 2022b)

#### 2.2.1 pH-responsive charge and/or hydrophilicity shifting systems

To equip carriers with pH-dependent activity, one strategy is to add moieties that can change charge and/or hydrophilicity when pH decreases. The acidic pH can trigger the protonation of those groups or the change from hydrophobicity to hydrophilicity of polymers, which leads to the degradation of carriers and the release of drugs (Deirram et al., 2019). In some cases, polymers are self-assembled into cationic hydrophilic exteriors and pH-responsive hydrophobic interiors. When pH decreases below their pKa, the inner groups become hydrophilic and the carriers cleave (Horev et al., 2015; Zhou et al., 2016; Zhang et al., 2021).

A kind of 21 nm, self-assembly cationic nanoparticles encapsulating farnesols were designed to achieve pH-responsive drug release and selective oral biofilm disruption (Horev et al., 2015; Zhou et al., 2016; Sims et al., 2018). The nanoparticles, consisting of 2-(dimethylamino)ethyl methacrylate (DMAEMA), butyl methacrylate (BMA), and 2-propylacrylic acid (PAA) (p(DMAEMA)-b-p(DMAEMA-co-BMA-co-PAA)), overcame the hydrophobicity-related bad effect of farnesols in conventional treatments against oral biofilms. The nanoparticle generated higher binding affinities to pellicle and EPS at acidic pH than physiological conditions and enhanced the antibiofilm activity of farnesol *via* promoting drug localization. Farnesol release rate at pH 4.5 was twice as fast as the rate at pH 7.2, indicating that farnesol release was activated by acidic pH. Compared to solely farnesol, 4-fold enhancement was discovered in *S. mutans* biofilms disruption in drug-loaded nanoparticles group at pH 4.5. The drug-loaded nanoparticles group compromised the mechanical stability of biofilms, thus displaying more than 2-fold biofilm removal ability compared to free farnesol when exposed to shear stress (Horev et al., 2015).

Zhou et al. synthesized pH-activated, doxycycline (DOX)-loaded nanoparticles that contained N,N,N-trimethyl chitosan (TMC) and liposomes (Lips) (Zhou et al., 2018). The data displayed that DOX release half-life was 0.75 h at pH 4.5, yet 2.3 h at pH 6.8. In addition to releasing the DOX, TMC processed an antibacterial effect itself. Hu et al. tested the antibiofilm activity of this nanoparticle and results indicated that TMC-Lip-DOX nanoparticles disrupted the biofilm architecture and reduced the number of bacteria significantly, compared with TMC group and DOX group (Hu et al., 2019). Both *in vivo* and *in vitro* tests showed that the nanoparticles were able to inhibit dental plaque effectively and had nontoxicity.

Glycol chitosan with pH-activated charge inversion is able to target acidic bacterial infection sites and exhibits better antimicrobial efficiency (Yu et al., 2019; Yan et al., 2020). Based on this, a novel kind of photothermal antimicrobial nanoagent with pH responsiveness, named FePAgPG, was synthesized (Xu et al., 2022a). Fe_3_O_4_ nanoparticles were modified by Ag and two polydopamine layers in sequence and then wrapped with glycol chitosan. When pH decreased, the zeta potential of FePAgPG shifted from anionic (-24.57 ± 1.31 mV) to cationic value (7.89 ± 0.48 mV) due to the protonation of glycol chitosan. Thus, at acidic pH, the positively charged nanoparticles could better attach to negative *S. mutans* and 1.7-fold enhancements in efficacy against *S. mutans* was observed at pH 5.4 than at pH 7.4. *Via* infrared irradiation at low temperature, FePAgPG nanoparticles exerted a potent antimicrobial rate of over 95% against planktonic *S. mutans* and inhibited the formation of *S. mutans* biofilm.

Sun et al. designed a pH-responsive hydrogel coating which loaded antimicrobial peptides to capture and kill microbes. The pH-responsive coating, carboxybetaine methacrylate-dimethylaminoethyl methacrylate copolymer p(CBMA-co-DMAEMA), could capture bacteria and release antimicrobial peptides simultaneously, performing as a smart hunter (Sun et al., 2022). Zeta potentials of the surface shifted from -0.79 mV at pH 7 to 4.07 mV, 8.05 mV and 54.03 mV at pH 6, 5 and 4 respectively. When pH changed from 7.0 to 5.5, the number of dead bacteria on hydrogel layers loaded with antimicrobial peptides increase. The result indicated that the pH-activated capture of hydrogel layers mainly relied on cationic surface charge.

In order to inhibit cariogenic biofilms, Peng et al. designed a novel CHX-loaded pH-sensitive nanoparticle (p(DH)@CHX), composed of DMAEMA and hydroxyethyl methacrylate (HEMA) (Peng et al., 2022). According to data, the release of CHX was stable with low volume over time under physiological conditions, yet increased gradually at the acidic pH. In the test against cariogenic biofilm, researchers found that both p(DH)@CHX and CHX were capable of inhibiting the lactic acid production by biofilms, and no significant difference was found in the lactic acid production. CHX, an antibacterial agent with pronounced cytotoxicity while continuously used, exerted lower cytotoxicity against human oral keratinocytes when loaded into nanoparticles. Besides, p(DH)@CHX showed no effect on healthy saliva-derived biofilm while exhibiting the same antimicrobial activities as free CHX on cariogenic biofilm.

An antibacterial agent, bedaquiline, exhibits high effects on killing planktonic bacteria but have limited efficacy in removing mature biofilm (Flemming et al., 2016; Bowen et al., 2018; Zhang et al., 2021). In order to improve permeability of bedaquiline to mature biofilm, Zhang et al. synthesized a novel pH-activated nano micelle, core-shell nano micelle (mPEG-b-PDPA), for loading hydrophobic antibacterial agents (Zhang et al., 2021). The release rate of bedaquiline was very gentle at pH 7, with about 35% in the first 12 h, while the amount released within 3 h reached 92.2% at pH 5. It has been confirmed that the bedaquiline-loaded micelles system could inhibit *S. mutans* biofilm formation and take antimicrobial effect against mature *S. mutans* biofilm.

#### 2.2.2 pH-responsive systems with acid-degradable linkages

A simple and compelling strategy to design pH responsive polymers is to design nanocarriers including pH-responsive linkages with stabilization at neutral pH and activity at acidic pH. Those linkages mainly incorporate hydrazone linkages with ketone/aldehyde and hydrazide; imine linkages with an aldehyde (ketone) and amine; maleic acid amide linkages with amine and maleic anhydride; ortho ester linkages with alcohols and formate or ester (Deirram et al., 2019).

Aimed at reducing the side effect caused by the low selectivity of CHX, Zhao et al. designed a type of pH-responsive polymer that could release CHX in acid niches of cariogenic biofilms (Zhao et al., 2019). The whole system was named CA-PICMs. CHX was encapsulated in the core-shell polyionic complex micelles (PICMs) which were composed of cationic poly(ethylene glycol)-block-poly(2-((2-aminoethyl)carbamoyl)oxy)ethyl methacrylate (PEG-b-PAECOEMA) and anionic citraconic anhydride (CA). The citraconic amide is acid-degradable, and PEG block promotes the stability of the structure regardless of enzyme, pH, and temperature (Harris and Chess, 2003; Osada et al., 2005). While CA-PICMs reduced the toxicity of CHX, there was no statistical difference in antibacterial effects against *S. mutans* biofilms between CA-PICMs and CHX, which indicates that the polymers may be a promising approach for dental caries therapy (Zhao et al., 2019).

In order to combat tooth decay and enhance enamel restoration, Xu et al. synthesized the micellars, 3-maleimidopropionic acid-poly(ethyleneglycol)-block-poly(L-lysine)/phyenylboronic acid (MAL-PEG-b-PLL/PBA), which contained pH-cleavable boronate ester bond (Xu et al., 2022b). The antibacterial agent tannic acid, NaF, and salivary-acquired peptide (SAP) were conjugated with MAL-PEG-b-PLL/PBA to form PMs@NaF-SAP. PMs@NaF-SAP exerted better performance against *S. mutans* biofilm under acidic environment, since there was an increase in drug release when pH dropped. When pH reached 5, the antibacterial potency of PMs@NaF-SAP was stronger than the positive control treatment with CHX. In addition, PMs@NaF-SAP showed a significant inhibitory on bacterial adhesion compared with PMs@NaF, whilst NaF added inhibited demineralization and facilitated remineralization in enamel slices.

Farnesal (Far) was conjugated to PEG *via* acid-sensitive hydrazone bonds, which was then linked with pyrophosphate (PPi) and encapsulated into polymeric micelles to form a novel drug delivery system, PPi-Far-PMs (Yi et al., 2020). Far was released from its carriers much faster under an acidic condition (pH 4.5) than in a neutral environment (pH 7.4). PPi-Far-PMs could bind to dental enamel rapidly and inhibit the growth of *S. mutans*, while the antibacterial effects of free Far and farnesol groups showed no obvious difference from negative control. The *in vivo* test showed that PPi-Far-PMs facilitated the antibiofilm ability of Far, as well as restoring the microarchitecture of teeth with caries.

Yue et al. synthesized a novel kind of mesoporous silica nanoparticles (MSNs) with disulfide bonds introduced into the silica framework, which improved the degradable ability faced with environmental stimuli (Yue et al., 2018). To battle oral pathogens, Lu et al. loaded these MSNs with silver and CHX to form a novel kind of redox/pH dual-controlled nanoparticles (Lu et al., 2018). It has been confirmed that Ag-MSNs@CHX exhibited a glutathione- and pH-dependent release behavior of silver ions and CHX. Compared with CHX, Ag-MSNs@CHX exerted a more effective ability to inhibit the growth of *S. mutans* biofilms.

#### 2.2.3 Others

Fenton reaction is a classical reaction that catalyzes H_2_O_2_ to generate strong oxidizing hydroxyl radical (•OH) and other reactive oxygen species under the effect of ferrous ion (Fe^2+^) (Pignatello et al., 2006). It has been widely used to degrade organic matter that is difficult to be removed in sewage. In recent years, Fenton and Fenton-like reactions have been applied to other fields beyond the ecological environment, such as Chemodynamic therapy (CDT). CDT is a novel strategy to induce the apoptosis of cancer cells *via* catalyzing H_2_O_2_ to produce •OH and other strong oxidizing active species in the acidic microenvironment of tumor lesion areas (Li et al., 2021b). Since the pH of the cariogenic microenvironment is below physiological pH and ROS is capable of killing bacteria, researchers applied Fenton and Fenton-like reactions to antibiofilm. Based on Fenton and Fenton-like reaction, novel copper-doped zinc peroxide nanoparticles with the antibacterial agent Rose Bengal (ZnO_2_-Cu@RB NPs) were created (Zhang et al., 2022b). H_2_O_2_ can be created by the reaction between ZnO_2_ and hydrogen ions (H^+^) in the acid environment, which triggers the Fenton-like reaction between Cu and H_2_O_2_. *In-vitro* results showed that ZnO_2_-Cu@RB NPs performed potent inhibition against *S.mutans* in acidic biofilm. Reduction in the demineralization of apatite and suppression in the formation of EPS could also be found *in vitro* studies.

## 3 Mechanisms of action

**Figure 2:**
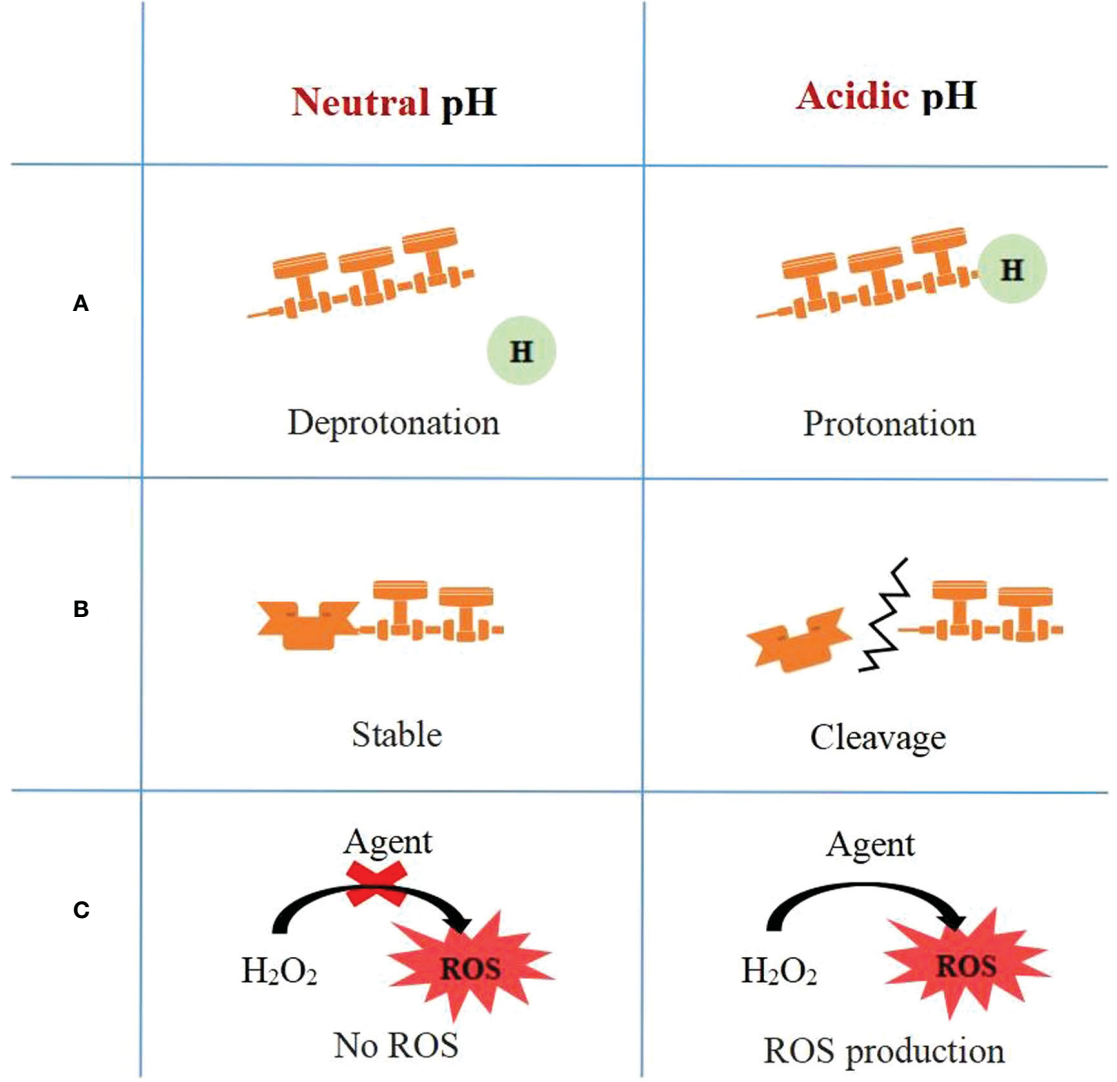
Mechanism of pH-activated antibacterial strategies: **(A)** Protonation and deprotonation reaction, **(B)** Acid labile linkages and **(C)** H^+^-triggered ROS production.

Many antibacterial mechanisms of pH-responsive antibacterial strategies have been proposed, and can be summarized into the following three parts: (1) Protonation and deprotonation reactions. Protonation reactions can transform the charge and structure of antimicrobial agents or drug carriers under an acid environment. (2) Acid labile linkages. Those linkages can cleave at low pH, contributing to the disassembly of carriers and drug release. (3) H^+^-triggered ROS production. By generating ROS under low pH, ROS productive systems can kill bacteria in cariogenic acidic biofilm selectively with slight side effects (Deirram et al., 2019; Fu et al., 2021) (**Figure 2**).

### 3.1 Protonation and deprotonation reactions

Protonation is capturing protons to chemical species, such as atoms, molecules, or ions at low pH, while deprotonation is removing protons when pH increases. Properties of chemicals, such as charge and hydrophilic, change after protonation or deprotonation of specific groups. The low pH value in cariogenic microenvironment can trigger the protonation of chemicals and then result in the transformation of electric properties. In recent years, based on protonation and deprotonation mechanism, researchers have explored novel pH-activated antibiofilm strategies for dental caries control.

*Via* protonation reaction, pH-responsive antimicrobial peptides can change their electric properties and structures under low pH conditions. Histidine residue is the pH-responsive reactive site of most AMPs. Histidine is a basic amino acid with an imidazole side chain that can rapidly accept or provide protons when pH changes (Horch et al., 2014; Chen et al., 2019). The pK_a_ of histidine is about 6.5, which is closest to the normal oral physiological pH among the pKa of the 20 proteinogenic amino acids. Histidine is uncharged with hydrophobicity at neutral pH but can be protonated and turned into a hydrophilic residue when the ambient pH below its pK_a_. Histidine has been widely utilized in the design of pH-sensitive antimicrobial peptides. *Via* the protonation of histidine residues at low pH, most antimicrobial peptides are positively charged and form an amphipathic structure. Positive charge enhances their binding ability to the anionic microbial cell membrane, and amphipathic structure facilitates the peptides to form pores in membrane bilayer or penetrate into cells to act on intracellular target points (Bechinger and Gorr, 2017). Hydrophobic surfaces help peptides insert into microbial membranes, mediating direct membrane disruption, which can be stabilized by the interaction between hydrophilic surfaces and the head group regions of the membranes *via* electrostatic adsorption (Thaker et al., 2013; Xiong et al., 2015). According to protonation reaction, scholars introduced histidine residues into antibacterial peptides to prepare pH-responsive antimicrobial peptide and carry out a series of studies.

Histatin-5 is one of eminent forms of human histatins, which has been reported to have potent activity against *Candida* organisms (Khurshid et al., 2017). Low pH can enhance the positive charge of histatin-5 *via* protonation of histidine residues, which facilitates the localization of histatin-5 on anionic membranes, thereby inducing perturbations on the cell surface that leads to a rapid translocation of the peptide into the cytoplasm (Mochon and Liu, 2008). Unlike typical pore-forming peptides, histatin-5 influences cell membrane functions by acting on intracellular targets. In addition, some researchers designed a novel pH-dependent peptide pHly-1 based on the structure of Lycosin-I, a cationic and amphiphilic peptide (Tan et al., 2013; Wang et al., 2014c). Aimed at enduing the peptide with the pH-responsive ability, researchers primarily exchanged lysine residues with histidine residues which increases positive charge and hydrophobicity of pHly-1; the anionic glutamic acid residues within lycosin-I were replaced by neutral glutamine residues which enabled pHly-1 to better interact with anionic microbial membranes; to improve hydrophobicity for membrane disruption, isoleucine with hydrophobicity substituted glycine and serine which are more hydrophilic (Zhang et al., 2022a). Especially, compared with the β-sheets and nanofibers structures at neutral pH, pHly-1 could adopt random coil-helix conformation and change into nanoparticles which promotes membrane permeation when pH decreased. Furthermore, inspired by the structure and pH-dependent ability of Clavanin A, other researchers designed two 14 aa long acid-activated peptides (AAPs) rich in histidine and phenylalanine residues to combat dental caries (Clark et al., 2021). *Via* protonation of histidine residues at low pH, positive charges of AAPs increased, and AAPs underwent a transition from a helical conformation to a random coil. Although the explicit antimicrobial mechanism of AAPs is still unclear, the study confirmed that antimicrobial activity was closely associated with the increase in positive charges. Moreover, a 12 amino peptide, named GH12, with a large proportion of histidine residues and leucine residues, was synthesized (Wang et al., 2017). The protonation of histidine residues at low pH increased net positive charge of GH12, which led to the accumulation of cationic GH12 on negatively charged bacterial surfaces. *Via* the protonation reaction of histidine residues and increase of hydrophobicity around tryptophan sites, GH12 formed an amphipathic α-helix structure and killed bacteria by forming pores on cell membranes (Jiang et al., 2021). Similar to GH12, LH12 contains a histidine-rich sequence and can form an amphipathic α-helix structure to perturb microbial membranes (Jiang et al., 2022). Results showed that *via* reducing the gene expression of lactate dehydrogenase, alpha-subunit of F-type ATPase and glucosyltransferase, LH12 could inhibit acid production and biofilm formation.

Second strategy based on the protonation and deprotonation mechanism is the application of amine groups that exert switchable protonation and deprotonation ability along with the transformation of pH (Gannimani et al., 2020). Due to their pH switchable ability, amine groups have been widely applied to the design of antimicrobial agents. For example, DMAEM and HMAEM, two novel kinds of tertiary amines, were incorporated into resin to modify its pH-responsive antibacterial property. DMAEM and HMAEM are composed of a long-chain alkane that could insert into bacterial membranes, a tertiary amine group, and a methacrylate-containing alkane. They have been confirmed to exhibit acid-activated antimicrobial and anticaries effects (Liang et al., 2020). In addition to the application in the design of antimrobial agents, amine groups can be ionizable moieties in drug delivery polymers, such as p(DMAEMA), trimethylamine, and polydopamine. Some researchers used poly(DMAEMA-co-HEMA) as a pH-sensitive drug carrier (Peng et al., 2022). DMAEMA contains tertiary amine with switchable protonation/deprotonation ability while responding to changes in pH (Brahim et al., 2003). Besides, the pKa of DMAEMA (around 7.5) is close to physiological pH. When pH is below the pKa, the monomer can be protonated and undergo structural changes, thus leading to swelling of the whole nanoparticle and delivery of drugs. HEMA, a hydrophilic polymer, can induce hydrophilicity on hydrophobic surfaces (Gulsen and Chauhan, 2005; Roointan et al., 2018). Moreover, amine groups in chitosan can be protonated when pH decreases (Yu et al., 2019; Yan et al., 2020). Researchers synthesized TMC-Lip nanoparticles consisting of negatively charged liposomes and positively charged TMC. Liposome, coated by TMC *via* electrostatic adsorption effect, was used to encapsulate DOX. The residual amines of TMC, as pH-responsive moieties, could protonate at low pH, which led to the charges of TMC-Lip nanoparticles shifting to the positive. The positive nanoparticles were able to selectively target anionic microbial cell surfaces and accumulate in biofilms. Besides, the protonation of TMC led to the instability of nanoparticles and drug release (Verheul et al., 2008; Mourya and Inamdar, 2009). Others grafted glycol chitosan with polydopamine which coated on the surface of nanoparticles. *Via* the protonation of amine groups in glycol chitosan at acid pH, the negatively charged nanoparticles turned positive, resulting in stronger adhesion with acidic biofilm (Xu et al., 2022a). Furthermore, some other researchers synthesized a pH-responsive core-shell nano micelle, mPEG-b-PDPA, which was capable of loading Bedaquiline. At low pH (below 6), the protonation of amine groups in PDPA segments shifted hydrophobicity in the core to hydrophilicity, and the spherical nanostructure of the micelle swelled and even disassembled, which resulted in drug release (Zhang et al., 2021).

Polymers with carboxyl groups, such as PAA and carboxybetaine methacrylate (CBMA), can also be protonated and applied to designs of drug release systems. Based on this, p(CBMA-co-DMAEMA) was fabricated as a pH-responsive hydrogel layer (Sun et al., 2022). Due to the protonation/deprotonation of carboxyl groups as pH changes, CBMA possesses the pH-activated property (Shao and Jiang, 2015). The protonation of amine groups and carboxyl groups in a carrier can exhibit synergistic pH-activated effects, so DMAEMA was incorporated as a synergistic pH switch. The protonation of the CBMA and DMAEMA at low pH accounted for the positive charges of hydrogel surface, which can capture negatively charged pathogens. With the transition of the layer’s charge, the structure disassembles, accompanied by the release of antibacterial octapeptides (Sun et al., 2022). It is worth mentioning that the octapeptide (Ac-Leu-Lys-Phe-Gln-Phe-His-Phe-Asp-NH2, IKFQFHFD) also processes pH-responsive properties (Wang et al., 2019). After the carboxylate groups were protonated, the IKFQFHFD formed a cationic amphiphilic structure similar to that of AMP, generating a potential pH-activated antimicrobial effect. In addition, other researchers designed the p(DMAEMA)-b-p(DMAEMA-co-BMA-co-PAA) that could self-assemble into ~21 nm cationic nanoparticles. The nanoparticle consisted of cationic coronas, p(DMAEMA), and p(DMAEMA-co-BMA-co-PAA) cores, which formed a structure with a hydrophilic surface and pH-activated hydrophobic interior. At acidic pH, DMAEMA and PAA residues protonated and the structure of carriers became unstable, accounting for drug release. It has been confirmed that cationic nanoparticles could selectively accumulate in the negatively charged bacterial biofilm surface and target anionic microorganisms (Ng et al., 2013).

### 3.2 Acid labile linkages

In addition to mechanisms based on protonation and deprotonation, pH-responsive antibiofilm systems with linkages that are labile at acidic environment are other promising strategies. pH responsive carriers incorporate citraconic amide, boric acid ester, hydrazone, disulfide bond, acid-base reaction, or ortho ester, into them as acid labile linkages, with which carriers are able to degrade when pH decreases (Huang et al., 2014; Lu et al., 2018; Deirram et al., 2019; Xu et al., 2022b).

Citraconic amide is formed by the reaction between citraconic anhydride and primary amines. At physiological pH, the citraconic amide is negatively charged and stable, but when pH decreases, it promptly cleaves back into the positively charged primary amine *via* degradation of linkage (Huang et al., 2014). When citraconic amide is incorporated into pH-sensitive drug delivery vehicles, the cleavage of citraconic amide at low pH can break the electrostatic balance and trigger the degradation of whole polymers (Lee et al., 2007; Lee et al., 2009), leading to drug release. Based on this mechanism, PEG-b-PAECOEMA/CA, the pH responsiveness of which comes from citraconic amide groups, was fabricated as a carrier of CHX (Zhao et al., 2019).

Boric acid ester bond, formed by the reaction between boric acid and hydroxy compound, is stable at physiological pH, but can degrade at low pH. MAL-PEG-b-PLL/PBA, the micelles carrier of tannic acid, conjugated tannic acid to PBA *via* boric acid ester bond. The micelles were pH-activated and capable of cleaving under cariogenic acidic conditions (Xu et al., 2022b). When the nanoparticles targeted the cariogenic dental plaque, boronate ester bonds responded to low pH and degraded, resulting in the release of tannic acid.

The hydrazone linkages are formed by condensation between hydrazide groups of carriers and aldehyde or ketones groups of the drugs (Sonawane et al., 2017). Those linkages can promptly hydrolysis when pH decreases below neutral physiological pH. Based on this, hydrazone linkages have been widely applied in conjugation between drugs and polymer backbones, aimed at reducing systemic toxicity by pH-triggered drug delivery (Yoshida et al., 2013). For example, Far, derived from farnesol, was linked to PEG *via* hydrazone bonds. In acidic cariogenic microenvironment, the cleavage of hydrazine linkages leads to rapid release of Far, which improve the selectivity of Far (Chen et al., 2013; Mazzoni et al., 2015).

Disulfide bonds can cleave while reacting with H^+^ and glutathione. Lu et al. introduced disulfide bonds into the silica framework of a novel kind of Mesoporous silica nanoparticles (MSNs), which was designed with redox/pH dual-controlled drug release ability (Lu et al., 2018). When pH decreases, the MSNs could degrade *via* the cleavage of disulfide bonds and glutathiones in the matrix could accelerate this process, resulting in the release of loaded Ag ions and CHX.

The chemical complexes assembled by the interaction of acid and base are unstable at low pH. Based on this, a novel kind of pH activated quaternary pyridinium salt was synthesized. The reversible control of antibacterial activity is achieved by acid-base interaction. Azo-QPS-C16 is a kind of antimicrobial agent. At physiological pH, alkaline triethanolamine interacted with weakly acid Azo-QPS-C16, and then two or more Azo-QPS-C16 assembled tightly into a sandwich stacking structure, which prevented Azo-QPS-C16 from exerting antibacterial efficiency. If pH decreases, the sandwich stacking structure will collapse and the Azo-QPS-C16 will come into effect. The active antimicrobial part of the Azo-QPS-C16 is the quaternary pyridinium salt and long alkyl chain, which can interact with the hydrophobic membrane and lyse the cell (Chen and Cooper, 2002). The quaternary pyridinium salt was able to adsorb on bacterial membranes by protonation in low pH while the long alkyl chain could insert into microbial membranes (Cheng et al., 2017; Gannimani et al., 2020).

### 3.3 H^+^-triggered ROS production

ROS, an umbrella term referring to oxygen species with high reactivity, includes hydrogen peroxide (H_2_O_2_), singlet oxygen (^1^O_2_), hydroxyl radical (•OH), and superoxide anion radical (O_2_•^-^) (Nosaka and Nosaka, 2017; Cao et al., 2021). Besides directly damaging lipids, proteins, and DNA, ROS can destroy microbial membranes and cause the leakage of intracellular substances, which eventually results in the death of the bacteria (Li et al., 2021a). In addition, ROS production, as a major sterilization strategy, has higher antimicrobial efficacy and can reduce resistance of bacteria in contrast with traditional sterilization methods (Fu et al., 2021; Zhu et al., 2021). Therefore, H^+^-triggered ROS production is a promising strategy for biofilm control.

CDT is a novel strategy to control cariogenic biofilm *via* ROS. The acid microenvironment can be used as a stimulus to trigger the production of H_2_O_2_. Metals ions such as Fe^2+^, Mn^2+^, and Cu^2+^ are able to react with H_2_O_2_ and promote the accumulation of ROS, mainly •OH, through the Fenton reaction or Fenton-like reaction (Bokare and Choi, 2014; Tang et al., 2019; Zhou et al., 2021), which leads to degradation of refractory organics (Chamarro et al., 2001; Pignatello et al., 2006) and inhibition of biofilm growth (Gao et al., 2016). ZnO_2_-Cu@RB NPs are drug-loaded nanoparticles which achieves antibacterial responsiveness *via* the Fenton-like reaction (Zhang et al., 2022b). ZnO_2_ was added to produce H_2_O_2_ under acid environment by reaction ZnO_2_+2H^+^ → Zn^2+^+H_2_O_2_, enduing the nanoparticles with pH responsiveness. After that, copper ions could convert H_2_O_2_ into •OH with antibiofilm effects through the Fenton-like reaction (2Cu^2+^+H_2_O_2_ → 2Cu^+^+O_2_+2H^+^, Cu^+^+H_2_O_2_ → Cu^2+^+ OH^—^ +·OH).

CAT-NP can also produce ROS to control biofilms. Instead of relying on Fenton reactions, the catalytic activity has been confirmed to derive from the nanoparticles themselves (Wang et al., 2014a; Wang et al., 2014b). CAT-NP is able to perform a peroxidase-like activity that promptly catalyzes H_2_O_2_ at acid pH to form free radicals, which can both degrade EPS and kill bacteria (Gao et al., 2007). Aimed at enhancing the antibiofilm efficacy of iron oxide nanoparticles, some other researchers added GOx into iron oxide nanoparticles (Huang et al., 2021). GOx can catalyze glucose in EPS into H_2_O_2_ and H_2_O_2_ produced can react with iron oxide nanoparticles to produce ROS.

## 4 Biological safety

CHX is commonly used in clinical practice to prevent caries, but studies have shown that CHX has time-dependent and dose-dependent cytotoxic effects on gingival fibroblasts, and the concentration of 0.2% shows high toxicity. In addition, it kills oral microorganisms without selectivity and reduces the diversity of oral microbial community, thus destroying the ecological balance of microbial community and bringing great difficulties and challenges for clinical treatment (Mao et al., 2022). Studies have reported that pH-activated release of CHX and Ag-NPs biodegradable nanosystems (Ag-MSNs@CHX) can not only improve the anti-biofilm effect, compared to the CHX group showed significant cytotoxicity, Ag-MSNs@CHX also has a good safety at high concentrations (Lu et al., 2018). The smart pH-responsive agent, which only exerts antimicrobial action at acidic pH, is well suited for use in the uniquely acidic environment in which caries develop. It shows antibacterial effects during microbial dysregulation, rather than continuously killing all microorganisms, improving drug availability and maintaining microecological balance (Liang et al., 2020). pH-responsive drugs with targeted effects play their unique advantages in different fields, and their biocompatibility has attracted a lot of attention from scholars.

Many studies have designed pH activated antibacterial peptides, among which some scholars have reported that the antibacterial peptide GH12 has pH response characteristics, and this peptide shows stronger antibacterial and anti-biofilm activities under acidic pH. The peptide can not only maintain good stability in saliva, but also showed only a mild inhibitory effect at concentrations up to 128.0 μg/ml in a biotoxicity study, indicating a low cytotoxicity against human gingival fibroblasts (Jiang et al., 2021). Dual-sensitive antimicrobial peptide nanoparticles, pHly-1 NPs, showed reliable antimicrobial activity against Streptococcus pyogenes in acidic solutions mainly through cell membrane disruption. Compared with the clinically used mouthwash CHX, the development of dental caries in rats could be effectively inhibited with this nanoparticle. Moreover, by *in vitro* toxicity analysis, CHX showed an IC_50_ value of 4.9 μM against human gingival fibroblasts, while pHly-1NPs exceeded 500μM. A concentration of 31.25 μM CHX induced approximately 40% of gastric-like organ death, but no significant effect was observed for treatments with pHly-1 NPs up to 500μM. It was shown that the nanoparticles exhibited a higher safety profile compared to the clinically used antimicrobial agent CHX (Zhang et al., 2022a).

In addition to pH activated antibacterial peptides, a series of nanomaterials were developed and designed to degrade the biofilm matrix by catalyzing the *in situ* generation of free radicals from hydrogen peroxide in an acidic environment, thereby destroying the caries biofilm. It is worth mentioning that the bio-safety of the materials was also verified while testing their antibiofilm effect. The experimental results show that the pH-responsive nanohybrid particles exhibit strong catalytic activity and antibiofilm properties at acidic pH, which do not cause harmful effects on mucosal tissues such as gingiva and palate *in vivo* and have good biocompatibility (Gao et al., 2016; Naha et al., 2019; Huang et al., 2021; Zhang et al., 2022b).

To improve the targeted antibacterial ability and reduce the side effects of broad-spectrum antimicrobial agents, scholars developed targeted negatively charged doxycycline (DOX) loaded nanocarriers (TMC-Lip DOX NPs). The experimental results showed that the material was effective in pH-activated removal of cariogenic biofilms and was biocompatible with non-toxicity to MC3T3-E1 cells (Zhou et al., 2018). Furthermore, a reactive multidrug delivery system (PMs@NaF-SAP) has been reported to effectively inhibit biofilm formation, which specifically adheres to tooth, identifies cariogenic conditions and intelligently releases drugs at acidic pH, thereby providing cariogenic biofilm resistance and restoring the microarchitecture and mechanical properties of demineralized teeth. Toxicological analysis showed that the nanosystem had little to no adverse effects on cells as well as gingival and palatal tissues (Xu et al., 2022b). In summary, pH-responsive antimicrobial materials, which play an antimicrobial role intelligently only at acidic pH, have been shown to be stable and biocompatible, and are a promising anti-biofilm agent.

## 5 Limitation and future prospects

Although pH-activated strategies have been widely explored, there are still some challenges that remain to be overcome. First, the antibiofilm researches are limited to one or several pathogenic microbes. Dental plaque is a highly diverse community of microorganism, containing about 500 types of bacteria (Paster et al., 2001; Wong and Sissons, 2001; Rasiah et al., 2005), so it is provincial to examine antimicrobial efficiency of agents only by uncomplicated *in vitro* model. A more sophisticated biofilm model *in vitro* and animal caries model *in vivo* should be included to better predict the efficacy of antimicrobial agents in future.

Besides, mature biofilms are highly assembled microbial communities surrounded by extracellular matrix, which protects the resident in deep layer from the antibacterial medicine. Digesting the extracellular matrix helps to improve penetrability of medicine into mature biofilms. At present, some pH-activated antimicrobial medicine are capable of digesting extracellular matrix or inhibiting the formation of EPS, such as pHly-1, GH12, LH12, DMAEM@RA, ZnO2-Cu@RB NPs, and CAT-NP, but most of agents lack ability to digest extracellular matrix. Synergistic combination of pH-activated bacterial killing and EPS digestion is regarded as a promising direction because of targeting specificity and eliminating efficacy.

Furthermore, the physical and biological complexity of the oral environment, such as saliva, should be taken into consideration. Due to the rapid clearance of saliva, topically applied medicine usually shows poor retention and a temporary effect. It is of tremendous significance to study pH-activated strategies with a long-term antibacterial effect. Some researches endow medicines with stronger adhesive ability by adding components which can selectively adhere to dental enamel, such as SAP and tris(tetran-butylammonium) hydrogen pyrophosphate (Yi et al., 2020; Xu et al., 2022b). Therefore, improving adhesion of antimicrobial agents may be a promising direction.

The pH-activated drug delivery system is used for controlling antimicrobials release, but the systems exhibit an ephemeral effect since it is hard to recharge the loaded drug once released. Accordingly, it is vital to develop pH-activated drug delivery systems which can re-captured agents when pH increases.

Although the researches on pH-activated antibiofilm strategies are flourishing, they are just carried out *in vitro* or in animal studies. More studies including clinical trials are needed to facilitate the wider acceptance of pH-activated antibiofilm strategies for controlling dental caries.

## 6 Conclusion

With the enhancement of microbial balance, pH-activated therapeutics, precision-guided to the acidic niches, have become novel strategies to control dental caries and aroused increasing attention among researchers. There are of tremendous significance and application potential for pH-activated antibiofilm materials to be adopted for clinical dental applications. Many studies indicate that pH-activated antibiofilm materials will be beneficial in cariostatic filed. But before them entering the market and reaching the dental chair, huge challenges related to long-term effects and cost-effectiveness need to be conquered, and the *in-vivo* effect should be further validated in clinical experiments.

## Author contributions

XW, JL and SZ were involved in conceptualization, investigation, and writing original draft. WZ, LZ and XH were involved in review and editing. All authors contributed to the article and approved the submitted version.
